# 4′-Methyl-1*H*-14′,19′-dioxa-4′-aza­spiro­[indole-3,5′-tetra­cyclo­[18.4.0.0^2,6^.0^8,13^]tetra­cosa­ne]-1′(24′),8′,10′,12′,20′,22′-hexa­ene-2,7′(3*H*)-dione

**DOI:** 10.1107/S1600536812046132

**Published:** 2012-11-14

**Authors:** Sibi Narayanan, Thothadri Srinivasan, Santhanagopalan Purushothaman, Raghavachary Raghunathan, Devadasan Velmurugan

**Affiliations:** a Centre of Advanced Study in Crystallography and Biophysics, University of Madras, Guindy Campus, Chennai 600 025, India; b Department of Organic Chemistry, University of Madras, Guindy Campus, Chennai 600 025, India

## Abstract

In the title compound, C_29_H_28_N_2_O_4_, the indoline ring system is essentially planar, with a maximum deviation of 0.027 (2) Å; the carbonyl O atom lies 0.102 (1) Å out of the least-squares plane of the indole ring. The pyrrolidine ring adopts a C-envelope conformation, with a C atom displaced by 0.643 (2) Å from the mean plane formed by the remaining ring atoms. The pyrrolidine ring makes a dihedral angle of 86.1 (8)° with the indoline ring system. In the crystal, N—H⋯O hydrogen bonds result in the formation of cyclic centrosymmetric dimers [*R*_2_^2^(8)]. C—H⋯π inter­actions also occur, leading to a chain along the *b*-axis direction. There is a rather weak π–π electron inter­action between the pyrrazole and benzene rings, with a centroid–centroid distance of 3.765 (1) Å.

## Related literature

For background to natural and synthetic pharmacologically active pyrrolidines, see: Waldmann (1995 ▶). For related structures, see: Ganesh *et al.* (2012 ▶); Narayanan *et al.* (2012 ▶). For graph-set notation, see: Bernstein *et al.* (1995 ▶).
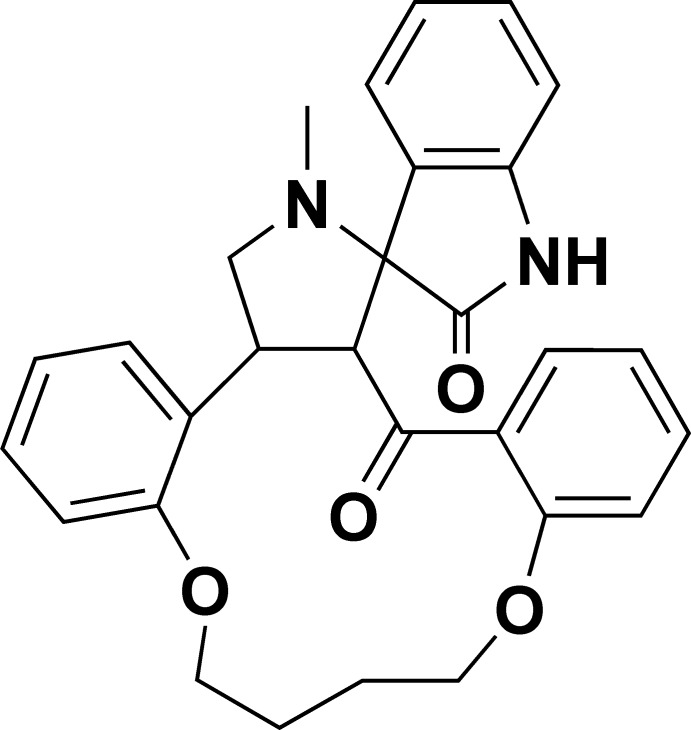


## Experimental

### 

#### Crystal data

C_29_H_28_N_2_O_4_
*M* *_r_* = 468.53
Triclinic, 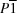

*a* = 9.4223 (3) Å
*b* = 10.5115 (3) Å
*c* = 14.1754 (5) Å
α = 70.235 (2)°
β = 87.309 (3)°
γ = 69.065 (2)°
*V* = 1229.67 (7) Å^3^
*Z* = 2
Mo *K*α radiation
μ = 0.09 mm^−1^
*T* = 293 K
0.25 × 0.22 × 0.19 mm

#### Data collection

Bruker APEXII CCD area detector diffractometer
Absorption correction: multi-scan (*SADABS*; Sheldrick, 2008 ▶) *T*_min_ = 0.979, *T*_max_ = 0.984
22180 measured reflections
5998 independent reflections
4260 reflections with *I* > 2σ(*I*)
*R*_int_ = 0.027

#### Refinement

*R*[*F* ^2^ > 2σ(*F* ^2^)] = 0.049
*wR*(*F* ^2^) = 0.147
*S* = 1.01
5998 reflections
317 parameters
H-atom parameters constrained
Δρ_max_ = 0.47 e Å^−3^
Δρ_min_ = −0.27 e Å^−3^

### 

Data collection: *APEX2* (Bruker, 2008 ▶); cell refinement: *SAINT* (Bruker, 2008 ▶); data reduction: *SAINT*; program(s) used to solve structure: *SHELXS97* (Sheldrick, 2008 ▶); program(s) used to refine structure: *SHELXL97* (Sheldrick, 2008 ▶); molecular graphics: *ORTEP-3* (Farrugia, 2012) ▶; software used to prepare material for publication: *SHELXL97* and *PLATON* (Spek, 2009 ▶).

## Supplementary Material

Crystal structure: contains datablock(s) global, I. DOI: 10.1107/S1600536812046132/pv2600sup1.cif

Structure factors: contains datablock(s) I. DOI: 10.1107/S1600536812046132/pv2600Isup2.hkl

Additional supplementary materials:  crystallographic information; 3D view; checkCIF report

## Figures and Tables

**Table 1:** Hydrogen-bond geometry (Å, °) *Cg*4 is the centroid of the C14–C19 ring.

*D*—H⋯*A*	*D*—H	H⋯*A*	*D*⋯*A*	*D*—H⋯*A*
N2—H2*A*⋯O2^i^	0.86	1.96	2.8105 (17)	170
C26—H26⋯*Cg*4^ii^	0.93	2.91	3.617 (3)	134

Symmetry codes: (i) 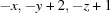; (ii) 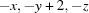.

**Fig. 1.**
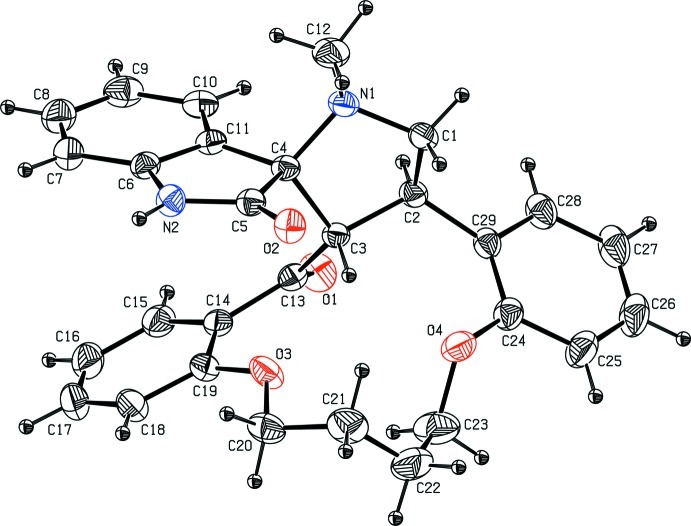
The molecular structure of the title compound, showing displacement ellipsoids drawn at the 30% probability level. H atoms are presented as small spheres of arbitrary radius.

**Fig. 2.**
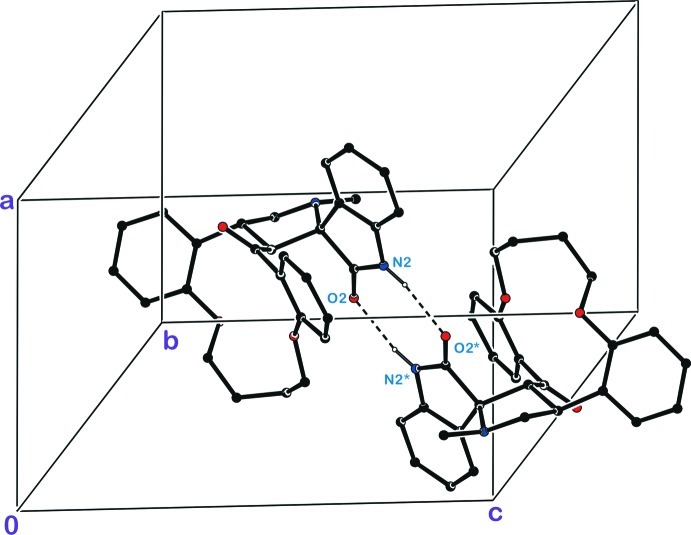
The crystal structure showing the formation of the centrosymmetric R22(8) dimer;; H atoms not involved in hydrogen bonding have been omitted for clarity. The dashed lines indicate hydrogen bonds. The atoms marked with an asterisk (*) are at the symmetry position (-x, 2 - y, 1 - z).

## supplementary crystallographic information

### Comment

Highly functionalized pyrrolidines have gained much interest in the past few years as they constitute main structural unit of many natural and synthetic pharmacologically active compounds (Waldmann, 1995). In continuation of our work on the crystal structure analysis of spiro-pyrrolidine derivatives (Narayanan *et al.*, 2012), the crystal structure of the title compound has been carried out and the results are presented here.

The bond lengths and angles in the title molecule (Fig. 1) are within normal ranges and comparable to those found in a related structure (Ganesh *et al.*, 2012). The indoline ring system (C4–C11/N2) is essentially planar, with maximum deviation of 0.027 (2) Å for atom C5; O2 lies 0.102 (1) Å out of the leastsquares plane of the indole ring. The pyrrolidine ring (C1–C4/N1) adopts a C1-envelop conformation with C1 0.643 (2) Å displaced from the mean-plane formed by the remaining ring atoms. The pyrrolidine ring makes a dihedral angle of 86.1 (8)° with the indoline ring system. The dihedral angle between the mean-planes of the pyrrolidine ring and the benzene ring (C24—C29) is 64.1 (1)°.

The crystal packing is stabilized by N—H···O, C—H···π and π–π interactions. N2—H2A···O2 hydrogen bonding results in a cyclic centrosymmetric dimer in *R*_2_^2^(8) ring motif (Bernstein *et al.*, 1995). There is a rather weak π–π electron interaction between the centroids of the pyrrazole (N2/C4/C5/C6/C11) and benzene (C14—C19) rings (*Cg*2···*Cg*4, respectively) with the centroid-centroid distance 3.765 (1) Å.

### Experimental

A mixture of isatin (150 mg, 1 mmol), sarcosine (90 mg, 1 mmol) and (4E)-12,17-dioxatricyclo[16.4.0.0^6,11^]docosa-1(22),4,6,8,10,18, 20-heptaen-3-one (300 mg 1.0 mmol) in toluene (20 ml) was refluxed under Dean-Stark reaction condition until the disappearance of starting materials as evidenced by TLC. The reaction mixture was concentrated in *vacuo* and extracted with water (50 ml) and dichloromethane (2x50 ml). The organic layer was washed with brine solution, dried with anhydrous sodium sulfate and concentrated in *vacuo*. The residue was purified by column chromatography with hexane-ethylacetate (9:1) mixture to yield macrocycle in good yields. The product was dissolved in ethylacetate and heated for two minutes. The resulting solution was subjected to crystallization by slow evaporation of the solvent resulting in single crystals suitable for XRD studies.

### Refinement

All H atoms were fixed geometrically and allowed to ride on their parent C atoms, with N—H = 0.86 Å and C—H distances in the range 0.93–0.98 Å with *U*_iso_(H) = 1.5*U*_eq_(methyl C) and 1.2*U*_eq_(non-methyl C/N).

### Crystal data

**Table:**

C_29_H_28_N_2_O_4_	*Z* = 2
*M**_r_* = 468.53	*F*(000) = 496
Triclinic, *P*1	*D*_x_ = 1.265 Mg m^−^^3^
Hall symbol: -P 1	Mo *K*α radiation, λ = 0.71073 Å
*a* = 9.4223 (3) Å	Cell parameters from 5998 reflections
*b* = 10.5115 (3) Å	θ = 1.5–28.3°
*c* = 14.1754 (5) Å	µ = 0.09 mm^−^^1^
α = 70.235 (2)°	*T* = 293 K
β = 87.309 (3)°	Block, colorless
γ = 69.065 (2)°	0.25 × 0.22 × 0.19 mm
*V* = 1229.67 (7) Å^3^	

### Data collection

**Table:**

Bruker APEXII CCD area detector diffractometer	5998 independent reflections
Radiation source: fine-focus sealed tube	4260 reflections with *I* > 2σ(*I*)
Graphite monochromator	*R*_int_ = 0.027
ω and φ scans	θ_max_ = 28.3°, θ_min_ = 1.5°
Absorption correction: multi-scan (*SADABS*; Sheldrick, 2008)	*h* = −12→12
*T*_min_ = 0.979, *T*_max_ = 0.984	*k* = −13→12
22180 measured reflections	*l* = −18→18

### Refinement

**Table:**

Refinement on *F*^2^	Primary atom site location: structure-invariant direct methods
Least-squares matrix: full	Secondary atom site location: difference Fourier map
*R*[*F*^2^ > 2σ(*F*^2^)] = 0.049	Hydrogen site location: inferred from neighbouring sites
*wR*(*F*^2^) = 0.147	H-atom parameters constrained
*S* = 1.01	*w* = 1/[σ^2^(*F*_o_^2^) + (0.0689*P*)^2^ + 0.3628*P*] where *P* = (*F*_o_^2^ + 2*F*_c_^2^)/3
5998 reflections	(Δ/σ)_max_ < 0.001
317 parameters	Δρ_max_ = 0.47 e Å^−^^3^
0 restraints	Δρ_min_ = −0.27 e Å^−^^3^

### Special details

**Table:**

Geometry. All e.s.d.'s (except the e.s.d. in the dihedral angle between two l.s. planes) are estimated using the full covariance matrix. The cell e.s.d.'s are taken into account individually in the estimation of e.s.d.'s in distances, angles and torsion angles; correlations between e.s.d.'s in cell parameters are only used when they are defined by crystal symmetry. An approximate (isotropic) treatment of cell e.s.d.'s is used for estimating e.s.d.'s involving l.s. planes.
Refinement. Refinement of *F*^2^ against ALL reflections. The weighted *R*-factor *wR* and goodness of fit *S* are based on *F*^2^, conventional *R*-factors *R* are based on *F*, with *F* set to zero for negative *F*^2^. The threshold expression of *F*^2^ > σ(*F*^2^) is used only for calculating *R*-factors(gt) *etc*. and is not relevant to the choice of reflections for refinement. *R*-factors based on *F*^2^ are statistically about twice as large as those based on *F*, and *R*- factors based on ALL data will be even larger.

### Fractional atomic coordinates and isotropic or equivalent isotropic displacement parameters (Å^2^)

**Table:**

	*x*	*y*	*z*	*U*_iso_*/*U*_eq_	
C1	0.44418 (17)	0.81009 (18)	0.29447 (12)	0.0442 (4)	
H1A	0.3946	0.7414	0.3256	0.053*	
H1B	0.5502	0.7575	0.2883	0.053*	
C2	0.36234 (16)	0.91660 (17)	0.19251 (11)	0.0390 (3)	
H2	0.4231	0.9763	0.1617	0.047*	
C3	0.21938 (15)	1.01176 (16)	0.22618 (10)	0.0348 (3)	
H3	0.1490	0.9592	0.2438	0.042*	
C4	0.27685 (16)	1.01883 (17)	0.32641 (11)	0.0363 (3)	
C5	0.16358 (16)	0.99095 (17)	0.40634 (11)	0.0388 (3)	
C6	0.16331 (17)	1.21653 (18)	0.38333 (11)	0.0413 (3)	
C7	0.1279 (2)	1.3514 (2)	0.39076 (15)	0.0548 (4)	
H7	0.0538	1.3844	0.4314	0.066*	
C8	0.2075 (2)	1.4365 (2)	0.33506 (16)	0.0624 (5)	
H8	0.1861	1.5284	0.3383	0.075*	
C9	0.3177 (2)	1.3870 (2)	0.27514 (16)	0.0591 (5)	
H9	0.3700	1.4456	0.2389	0.071*	
C10	0.35165 (19)	1.25080 (19)	0.26815 (13)	0.0483 (4)	
H10	0.4261	1.2177	0.2277	0.058*	
C11	0.27254 (16)	1.16546 (17)	0.32249 (11)	0.0391 (3)	
C12	0.4820 (2)	0.8338 (2)	0.45746 (14)	0.0600 (5)	
H12A	0.4254	0.7730	0.4891	0.090*	
H12B	0.4661	0.9054	0.4886	0.090*	
H12C	0.5886	0.7756	0.4648	0.090*	
C13	0.13484 (17)	1.15881 (17)	0.14799 (11)	0.0410 (3)	
C14	−0.01066 (18)	1.25884 (17)	0.17087 (12)	0.0431 (4)	
C15	−0.0379 (2)	1.4067 (2)	0.13226 (14)	0.0559 (5)	
H15	0.0331	1.4388	0.0936	0.067*	
C16	−0.1679 (3)	1.5065 (2)	0.15010 (19)	0.0705 (6)	
H16	−0.1857	1.6052	0.1224	0.085*	
C17	−0.2714 (3)	1.4584 (2)	0.20962 (18)	0.0714 (6)	
H17	−0.3574	1.5253	0.2238	0.086*	
C18	−0.2492 (2)	1.3136 (2)	0.24813 (15)	0.0605 (5)	
H18	−0.3201	1.2826	0.2878	0.073*	
C19	−0.12012 (18)	1.21319 (18)	0.22752 (13)	0.0465 (4)	
C20	−0.2101 (2)	1.0135 (2)	0.29678 (16)	0.0590 (5)	
H20A	−0.3031	1.0661	0.2523	0.071*	
H20B	−0.2321	1.0224	0.3623	0.071*	
C21	−0.1499 (2)	0.8579 (2)	0.30534 (19)	0.0699 (6)	
H21A	−0.0474	0.8133	0.3382	0.084*	
H21B	−0.2122	0.8103	0.3488	0.084*	
C22	−0.1449 (3)	0.8287 (4)	0.2094 (2)	0.0923 (8)	
H22A	−0.1226	0.7257	0.2263	0.111*	
H22B	−0.2466	0.8790	0.1751	0.111*	
C23	−0.0380 (3)	0.8677 (3)	0.13700 (17)	0.0814 (7)	
H23A	−0.0434	0.8353	0.0812	0.098*	
H23B	−0.0684	0.9724	0.1105	0.098*	
C24	0.2312 (2)	0.78928 (19)	0.11634 (12)	0.0484 (4)	
C25	0.2379 (3)	0.7110 (2)	0.05322 (15)	0.0645 (5)	
H25	0.1613	0.6753	0.0513	0.077*	
C26	0.3570 (3)	0.6860 (2)	−0.00634 (15)	0.0742 (7)	
H26	0.3613	0.6325	−0.0476	0.089*	
C27	0.4695 (3)	0.7399 (2)	−0.00493 (15)	0.0709 (6)	
H27	0.5494	0.7241	−0.0458	0.085*	
C28	0.4633 (2)	0.8177 (2)	0.05755 (13)	0.0553 (5)	
H28	0.5396	0.8544	0.0577	0.066*	
C29	0.34579 (18)	0.84291 (17)	0.12054 (11)	0.0424 (4)	
N1	0.43033 (14)	0.90587 (15)	0.35091 (10)	0.0425 (3)	
N2	0.10136 (15)	1.11034 (15)	0.43241 (10)	0.0443 (3)	
H2A	0.0322	1.1202	0.4740	0.053*	
O1	0.18613 (16)	1.19738 (15)	0.06779 (9)	0.0620 (4)	
O2	0.13719 (13)	0.87891 (13)	0.44025 (9)	0.0485 (3)	
O3	−0.09428 (12)	1.06941 (13)	0.25689 (11)	0.0578 (3)	
O4	0.11530 (14)	0.80546 (15)	0.18039 (10)	0.0580 (3)	

### Atomic displacement parameters (Å^2^)

**Table:**

	*U*^11^	*U*^22^	*U*^33^	*U*^12^	*U*^13^	*U*^23^
C1	0.0335 (7)	0.0489 (9)	0.0462 (9)	−0.0082 (6)	0.0004 (6)	−0.0184 (7)
C2	0.0332 (7)	0.0465 (8)	0.0389 (8)	−0.0142 (6)	0.0068 (6)	−0.0173 (6)
C3	0.0308 (6)	0.0425 (8)	0.0331 (7)	−0.0142 (6)	0.0034 (5)	−0.0146 (6)
C4	0.0322 (7)	0.0450 (8)	0.0327 (7)	−0.0149 (6)	0.0035 (5)	−0.0138 (6)
C5	0.0373 (7)	0.0472 (9)	0.0332 (7)	−0.0164 (6)	0.0040 (6)	−0.0145 (6)
C6	0.0411 (8)	0.0470 (9)	0.0372 (8)	−0.0161 (6)	−0.0008 (6)	−0.0155 (7)
C7	0.0540 (10)	0.0516 (10)	0.0590 (11)	−0.0134 (8)	0.0004 (8)	−0.0249 (9)
C8	0.0652 (12)	0.0455 (10)	0.0748 (13)	−0.0191 (9)	−0.0087 (10)	−0.0177 (9)
C9	0.0566 (10)	0.0537 (11)	0.0650 (12)	−0.0298 (9)	−0.0047 (9)	−0.0060 (9)
C10	0.0421 (8)	0.0575 (10)	0.0457 (9)	−0.0239 (7)	0.0010 (7)	−0.0118 (8)
C11	0.0362 (7)	0.0476 (9)	0.0349 (7)	−0.0173 (6)	−0.0013 (6)	−0.0131 (6)
C12	0.0524 (10)	0.0723 (13)	0.0457 (10)	−0.0122 (9)	−0.0117 (8)	−0.0177 (9)
C13	0.0430 (8)	0.0452 (9)	0.0363 (8)	−0.0167 (7)	−0.0014 (6)	−0.0146 (7)
C14	0.0421 (8)	0.0417 (8)	0.0416 (8)	−0.0087 (6)	−0.0092 (6)	−0.0149 (7)
C15	0.0617 (11)	0.0467 (10)	0.0551 (10)	−0.0153 (8)	−0.0118 (8)	−0.0146 (8)
C16	0.0753 (14)	0.0438 (10)	0.0839 (15)	−0.0058 (10)	−0.0191 (12)	−0.0251 (10)
C17	0.0628 (12)	0.0596 (13)	0.0836 (15)	0.0037 (10)	−0.0081 (11)	−0.0403 (11)
C18	0.0449 (9)	0.0651 (12)	0.0643 (12)	−0.0035 (8)	−0.0008 (8)	−0.0303 (10)
C19	0.0382 (8)	0.0463 (9)	0.0491 (9)	−0.0060 (7)	−0.0055 (7)	−0.0181 (7)
C20	0.0375 (8)	0.0751 (13)	0.0644 (12)	−0.0230 (8)	0.0119 (8)	−0.0223 (10)
C21	0.0538 (11)	0.0755 (14)	0.0874 (16)	−0.0374 (10)	0.0172 (10)	−0.0230 (12)
C22	0.0600 (13)	0.130 (2)	0.128 (2)	−0.0507 (15)	0.0145 (14)	−0.078 (2)
C23	0.0672 (13)	0.122 (2)	0.0594 (13)	−0.0371 (14)	−0.0041 (10)	−0.0318 (13)
C24	0.0565 (10)	0.0463 (9)	0.0401 (8)	−0.0138 (7)	−0.0013 (7)	−0.0165 (7)
C25	0.0839 (14)	0.0582 (11)	0.0545 (11)	−0.0209 (10)	−0.0098 (10)	−0.0262 (9)
C26	0.1009 (17)	0.0651 (13)	0.0479 (11)	−0.0059 (12)	−0.0087 (11)	−0.0327 (10)
C27	0.0766 (14)	0.0761 (14)	0.0452 (10)	−0.0013 (11)	0.0078 (9)	−0.0313 (10)
C28	0.0539 (10)	0.0605 (11)	0.0422 (9)	−0.0077 (8)	0.0070 (7)	−0.0206 (8)
C29	0.0443 (8)	0.0416 (8)	0.0350 (8)	−0.0073 (6)	0.0018 (6)	−0.0144 (6)
N1	0.0336 (6)	0.0514 (8)	0.0397 (7)	−0.0111 (5)	−0.0009 (5)	−0.0163 (6)
N2	0.0459 (7)	0.0519 (8)	0.0399 (7)	−0.0203 (6)	0.0139 (6)	−0.0205 (6)
O1	0.0688 (8)	0.0641 (8)	0.0401 (7)	−0.0195 (7)	0.0096 (6)	−0.0077 (6)
O2	0.0527 (7)	0.0497 (7)	0.0469 (6)	−0.0241 (5)	0.0162 (5)	−0.0168 (5)
O3	0.0342 (6)	0.0485 (7)	0.0844 (9)	−0.0120 (5)	0.0118 (6)	−0.0192 (6)
O4	0.0540 (7)	0.0760 (9)	0.0608 (8)	−0.0331 (6)	0.0075 (6)	−0.0345 (7)

### Geometric parameters (Å, º)

**Table:**

C1—N1	1.453 (2)	C15—C16	1.377 (3)
C1—C2	1.526 (2)	C15—H15	0.9300
C1—H1A	0.9700	C16—C17	1.380 (3)
C1—H1B	0.9700	C16—H16	0.9300
C2—C29	1.517 (2)	C17—C18	1.372 (3)
C2—C3	1.530 (2)	C17—H17	0.9300
C2—H2	0.9800	C18—C19	1.392 (2)
C3—C13	1.515 (2)	C18—H18	0.9300
C3—C4	1.5778 (19)	C19—O3	1.356 (2)
C3—H3	0.9800	C20—O3	1.426 (2)
C4—N1	1.4707 (18)	C20—C21	1.490 (3)
C4—C11	1.509 (2)	C20—H20A	0.9700
C4—C5	1.547 (2)	C20—H20B	0.9700
C5—O2	1.2227 (19)	C21—C22	1.488 (4)
C5—N2	1.350 (2)	C21—H21A	0.9700
C6—C7	1.376 (2)	C21—H21B	0.9700
C6—C11	1.388 (2)	C22—C23	1.466 (4)
C6—N2	1.405 (2)	C22—H22A	0.9700
C7—C8	1.390 (3)	C22—H22B	0.9700
C7—H7	0.9300	C23—O4	1.425 (2)
C8—C9	1.379 (3)	C23—H23A	0.9700
C8—H8	0.9300	C23—H23B	0.9700
C9—C10	1.388 (3)	C24—O4	1.387 (2)
C9—H9	0.9300	C24—C25	1.392 (2)
C10—C11	1.382 (2)	C24—C29	1.397 (2)
C10—H10	0.9300	C25—C26	1.375 (3)
C12—N1	1.457 (2)	C25—H25	0.9300
C12—H12A	0.9600	C26—C27	1.372 (3)
C12—H12B	0.9600	C26—H26	0.9300
C12—H12C	0.9600	C27—C28	1.381 (3)
C13—O1	1.211 (2)	C27—H27	0.9300
C13—C14	1.500 (2)	C28—C29	1.397 (2)
C14—C15	1.390 (2)	C28—H28	0.9300
C14—C19	1.398 (2)	N2—H2A	0.8600
			
N1—C1—C2	102.16 (13)	C15—C16—H16	120.4
N1—C1—H1A	111.3	C17—C16—H16	120.4
C2—C1—H1A	111.3	C18—C17—C16	121.00 (19)
N1—C1—H1B	111.3	C18—C17—H17	119.5
C2—C1—H1B	111.3	C16—C17—H17	119.5
H1A—C1—H1B	109.2	C17—C18—C19	119.7 (2)
C29—C2—C1	113.37 (13)	C17—C18—H18	120.2
C29—C2—C3	119.52 (12)	C19—C18—H18	120.2
C1—C2—C3	100.36 (12)	O3—C19—C18	123.51 (17)
C29—C2—H2	107.6	O3—C19—C14	116.14 (14)
C1—C2—H2	107.6	C18—C19—C14	120.32 (17)
C3—C2—H2	107.6	O3—C20—C21	106.46 (15)
C13—C3—C2	115.22 (12)	O3—C20—H20A	110.4
C13—C3—C4	114.19 (12)	C21—C20—H20A	110.4
C2—C3—C4	104.28 (11)	O3—C20—H20B	110.4
C13—C3—H3	107.6	C21—C20—H20B	110.4
C2—C3—H3	107.6	H20A—C20—H20B	108.6
C4—C3—H3	107.6	C22—C21—C20	116.2 (2)
N1—C4—C11	113.80 (12)	C22—C21—H21A	108.2
N1—C4—C5	114.13 (12)	C20—C21—H21A	108.2
C11—C4—C5	101.31 (12)	C22—C21—H21B	108.2
N1—C4—C3	103.47 (11)	C20—C21—H21B	108.2
C11—C4—C3	116.05 (12)	H21A—C21—H21B	107.4
C5—C4—C3	108.39 (11)	C23—C22—C21	119.24 (19)
O2—C5—N2	126.42 (14)	C23—C22—H22A	107.5
O2—C5—C4	125.33 (14)	C21—C22—H22A	107.5
N2—C5—C4	108.24 (13)	C23—C22—H22B	107.5
C7—C6—C11	122.42 (16)	C21—C22—H22B	107.5
C7—C6—N2	128.30 (16)	H22A—C22—H22B	107.0
C11—C6—N2	109.27 (14)	O4—C23—C22	112.6 (2)
C6—C7—C8	117.32 (18)	O4—C23—H23A	109.1
C6—C7—H7	121.3	C22—C23—H23A	109.1
C8—C7—H7	121.3	O4—C23—H23B	109.1
C9—C8—C7	121.11 (18)	C22—C23—H23B	109.1
C9—C8—H8	119.4	H23A—C23—H23B	107.8
C7—C8—H8	119.4	O4—C24—C25	119.57 (17)
C8—C9—C10	120.86 (18)	O4—C24—C29	119.84 (14)
C8—C9—H9	119.6	C25—C24—C29	120.44 (18)
C10—C9—H9	119.6	C26—C25—C24	120.5 (2)
C11—C10—C9	118.67 (17)	C26—C25—H25	119.7
C11—C10—H10	120.7	C24—C25—H25	119.7
C9—C10—H10	120.7	C27—C26—C25	120.12 (19)
C10—C11—C6	119.61 (16)	C27—C26—H26	119.9
C10—C11—C4	131.19 (15)	C25—C26—H26	119.9
C6—C11—C4	109.15 (13)	C26—C27—C28	119.6 (2)
N1—C12—H12A	109.5	C26—C27—H27	120.2
N1—C12—H12B	109.5	C28—C27—H27	120.2
H12A—C12—H12B	109.5	C27—C28—C29	122.0 (2)
N1—C12—H12C	109.5	C27—C28—H28	119.0
H12A—C12—H12C	109.5	C29—C28—H28	119.0
H12B—C12—H12C	109.5	C28—C29—C24	117.34 (16)
O1—C13—C14	119.75 (15)	C28—C29—C2	116.07 (15)
O1—C13—C3	120.24 (14)	C24—C29—C2	126.43 (14)
C14—C13—C3	119.99 (13)	C1—N1—C12	115.49 (14)
C15—C14—C19	118.30 (16)	C1—N1—C4	108.59 (11)
C15—C14—C13	117.35 (16)	C12—N1—C4	116.17 (13)
C19—C14—C13	124.35 (14)	C5—N2—C6	111.76 (13)
C16—C15—C14	121.4 (2)	C5—N2—H2A	124.1
C16—C15—H15	119.3	C6—N2—H2A	124.1
C14—C15—H15	119.3	C19—O3—C20	121.51 (14)
C15—C16—C17	119.2 (2)	C24—O4—C23	117.83 (15)
			
N1—C1—C2—C29	−174.22 (12)	C14—C15—C16—C17	1.6 (3)
N1—C1—C2—C3	−45.56 (14)	C15—C16—C17—C18	−2.3 (3)
C29—C2—C3—C13	−74.68 (18)	C16—C17—C18—C19	0.4 (3)
C1—C2—C3—C13	160.78 (13)	C17—C18—C19—O3	−175.51 (17)
C29—C2—C3—C4	159.35 (13)	C17—C18—C19—C14	2.3 (3)
C1—C2—C3—C4	34.82 (14)	C15—C14—C19—O3	175.02 (14)
C13—C3—C4—N1	−138.67 (13)	C13—C14—C19—O3	−4.5 (2)
C2—C3—C4—N1	−12.05 (15)	C15—C14—C19—C18	−2.9 (2)
C13—C3—C4—C11	−13.29 (17)	C13—C14—C19—C18	177.56 (15)
C2—C3—C4—C11	113.32 (14)	O3—C20—C21—C22	−73.7 (2)
C13—C3—C4—C5	99.82 (14)	C20—C21—C22—C23	68.2 (3)
C2—C3—C4—C5	−133.56 (13)	C21—C22—C23—O4	54.5 (4)
N1—C4—C5—O2	−51.95 (19)	O4—C24—C25—C26	−175.97 (17)
C11—C4—C5—O2	−174.67 (14)	C29—C24—C25—C26	−0.4 (3)
C3—C4—C5—O2	62.75 (18)	C24—C25—C26—C27	−0.8 (3)
N1—C4—C5—N2	127.34 (14)	C25—C26—C27—C28	0.8 (3)
C11—C4—C5—N2	4.62 (15)	C26—C27—C28—C29	0.4 (3)
C3—C4—C5—N2	−117.96 (13)	C27—C28—C29—C24	−1.6 (3)
C11—C6—C7—C8	−0.6 (3)	C27—C28—C29—C2	174.00 (16)
N2—C6—C7—C8	179.12 (15)	O4—C24—C29—C28	177.14 (15)
C6—C7—C8—C9	−0.2 (3)	C25—C24—C29—C28	1.6 (2)
C7—C8—C9—C10	0.5 (3)	O4—C24—C29—C2	2.0 (3)
C8—C9—C10—C11	0.1 (3)	C25—C24—C29—C2	−173.54 (16)
C9—C10—C11—C6	−0.8 (2)	C1—C2—C29—C28	−91.08 (17)
C9—C10—C11—C4	176.26 (15)	C3—C2—C29—C28	150.90 (15)
C7—C6—C11—C10	1.1 (2)	C1—C2—C29—C24	84.06 (19)
N2—C6—C11—C10	−178.63 (13)	C3—C2—C29—C24	−34.0 (2)
C7—C6—C11—C4	−176.56 (14)	C2—C1—N1—C12	172.65 (14)
N2—C6—C11—C4	3.68 (16)	C2—C1—N1—C4	40.12 (15)
N1—C4—C11—C10	54.8 (2)	C11—C4—N1—C1	−144.11 (13)
C5—C4—C11—C10	177.73 (15)	C5—C4—N1—C1	100.27 (15)
C3—C4—C11—C10	−65.1 (2)	C3—C4—N1—C1	−17.30 (16)
N1—C4—C11—C6	−127.89 (13)	C11—C4—N1—C12	83.73 (18)
C5—C4—C11—C6	−4.94 (14)	C5—C4—N1—C12	−31.89 (19)
C3—C4—C11—C6	112.18 (14)	C3—C4—N1—C12	−149.46 (14)
C2—C3—C13—O1	−4.4 (2)	O2—C5—N2—C6	176.50 (14)
C4—C3—C13—O1	116.34 (16)	C4—C5—N2—C6	−2.78 (17)
C2—C3—C13—C14	177.33 (13)	C7—C6—N2—C5	179.74 (16)
C4—C3—C13—C14	−61.97 (17)	C11—C6—N2—C5	−0.52 (17)
O1—C13—C14—C15	−34.4 (2)	C18—C19—O3—C20	10.7 (3)
C3—C13—C14—C15	143.95 (15)	C14—C19—O3—C20	−167.11 (16)
O1—C13—C14—C19	145.16 (17)	C21—C20—O3—C19	171.34 (17)
C3—C13—C14—C19	−36.5 (2)	C25—C24—O4—C23	−56.2 (3)
C19—C14—C15—C16	1.0 (3)	C29—C24—O4—C23	128.2 (2)
C13—C14—C15—C16	−179.46 (16)	C22—C23—O4—C24	157.44 (19)

### Hydrogen-bond geometry (Å, º)

Cg4 is the centroid of the C14–C19 ring.

**Table:**

*D*—H···*A*	*D*—H	H···*A*	*D*···*A*	*D*—H···*A*
N2—H2*A*···O2^i^	0.86	1.96	2.8105 (17)	170
C26—H26···*Cg*4^ii^	0.93	2.91	3.617 (3)	134

Symmetry codes: (i) −*x*, −*y*+2, −*z*+1; (ii) −*x*, −*y*+2, −*z*.
